# Clinical Lecture, in the U. S. Marine Hospital

**Published:** 1853-02

**Authors:** W. B. Herrick


*Clinical Lecture, in the U. S. Marine Hospital, by* W. B. Herrick, M. D.

The manner in which the tissues are nourished is somewhat obscure. If, however, we keep in mind what has been said of food and blood, and the classification that has been made, we shall be able to trace, to some extent at least, the steps in the physiological performance of the function of secondary assimilation, and to detect abnormal action. Since, in health, the supply of new material and the removal of old must be simultaneous—and since the diseases connected with the process of deposition of nutritious material are necessarily connected with, and modified by, primary excretion, we shall find it convenient to speak of these processes together.

When all the constituents of healthy blood, properly combined and in due proportions, are brought into contact in the capillary vessels with the tissues, there takes place a transfer of the albumen, probably in combination with oxygen and soda, into the tissues, where the soda combines with excrementitious acids formed by the oxygen uniting with the effete matter, and passes again into the blood-vessels, leaving the albumen deposited combined in some form with potash.

There are two classes of disease which seem to us to be dependent upon excess or deficiency of the mineral constituents of the blood. The one is developed in earlier life, during the growth of the osseous system; the other makes its appearance in more advanced years, when the demand for the earths especially is very much diminished. We refer to tuberculous and cancerous affections. In the wards of this hospital, during the earlier part of the winter, you have seen several cases of well-marked phthisis; and you will remember the treatment consisted of alkalies, the alkaline earths, lime especially, and cod-liver oil. Without entering into a minute examination of tuberculous matter, we may simply say that, in our opinion, the deposits depend mainly on an arrest of development, or organization, and the nutritious material passes into an excretable condition, without having subserved the purposes of the animal economy. In order to stimulate to organization, or to supply the formative forces, we need the mineral constituents of the blood. The ferro-phosphate of lime has been recently recommended by Dr. A. H. Ramsay.

The opposite condition is one which has not occurred in these wards, since the hospital was opened.

There is a class of patients now under treatment, which furnish interesting subjects of study. I allude to those affected with ulcers. You have, no doubt, observed a marked difference in these cases, — some presenting exuberant granulations of a pale-red color, with a tendency to rise above the surface, discharging thick cream-colored pus, with little tendency to cicatrize. Another class presents exactly the opposite characteristics,—the surface depressed below the surrounding parts, ragged edges, minute dark points studding the bottom and sides, with the exudation of a dark brownish sanious discharge very acrid and irritating to the surrounding tissues. You will recognise, in the first of these classes, exaggerated secondary assimilation and primary excretion; in the second, defective secondary assimilation, and, as we shall see presently, an imperfect performance of the function both of primary and secondary excretion. These two classes of ulcers are readily distinguished, and, as it seems to us, require different treatment. In the first, there is a tendency to too rapid cell growth, the granulations are pushed out from the living vascular tissues by a rapid formation in the effused plastic material, of new corpuscles; while the outer layer degenerates into pus, the quantity and quality depending upon the activity of the formative forces. Some of these cases are at times troublesome, but in these wards they are less frequently met with than those of the opposite class. In private practice, in patients free from any scrofulous or syphilitic taint, and especially where good diet and iron or other tonics are prescribed, you will almost always find this tendency to excessive granulation. The treatment required is mostly local. The application of nit. argent. to the surfaces, compression, and in some cases astringent washes. Little constitutional treatment is necessary, except, in cases where suppuration is extensive, a supporting diet, with mineral acids, which seem to us to favor oxidation. In the wards of this hospital there have been treated, during the last eight months, twenty patients for ulcers of legs alone. In addition to this, many of those who have been admitted for other diseases have been affected with ulcers. Of this number, we believe not more than three or four have presented, at the time of entering the hospital, the characters described above.

The second class presents a deficiency, to as great an extent as the other does a redundancy, of granulations. The small points on the excavated surface seem to be almost stationary, the effused fluid containing comparatively few exudation corpuscles, and being deficient in the protein compounds. From some observations which we have made, we think that in most cases the reaction is either acid or less alkaline than healthy pus. There is here evidently a want of the formative force, and relatively a deficiency of the alkalies or alkaline earths; secondary assimilation is imperfect; the broken down tissue is not built up; the excrementitious acids, for want of bases to combine with, are not excreted, and by their presence they act upon the surrounding tissues, as we believe, in a manner similar to that which takes place, when acids and protein compounds are brought in contact out of the system. It is true that the chemical force seems, in many instances, to be suspended in living organisms, and yet it cannot be destroyed; it still exists, and must act, although modified by perhaps other forces acting with it, and for a while, in some processes, even masking it entirely. The *inorganic* atom, with all its properties, is present in the *organic.*

The treatment of these cases, as indicated by the condition pointed out, will consist in the use of the bases, both alkaline and earthy, in combination with iodine, chlorine, or such acids as are readily excreted without the aid of oxygen, together with a generous nitrogenized diet. As local stimulants, we may use a weak solution of the bichloride of mercury, the protochloride in powder, and, in cases where the edges are indurated, blisters have been recommended—and, which seems to us a better treatment, the use of adhesive strap and bandages. By this means, the use of bandages, we are able to affect the capillary vessels in such a manner, that the blood is passed more rapidly through them, a greater quantity of the nutritious material is brought in contact with the solids, and the nutritive process, consisting in the deposition of new and the removal of old matter, becomes much more active. In addition to the general and local treatment of which we have spoken, rest, and an elevated position of the limb—if it be a lower extremity, as is the case in the patients before you—should be insisted on.

There is a class of ulcers which seems to partake of the nature of both those already noticed; the patient whom many of you saw in Ward 56, No. 2, will serve as an illustration. The granulations seemed to be formed rapidly, and yet the ulcers were not healing. If you examined carefully, you observed that the deep excavations did not present the appearance of healthy granulating surfaces. The little points were intensely red, but mixed with more or less grayish matter. They were painful; so much so, that the patient could get no rest day or night. There seems, in cases like this, to be an exaggeration of secondary assimilation and of primary excretion, or rather a too rapid destruction of the forming tissues, while the formative force itself is normal in quantity, but increased in intensity. The treatment in the case to which we have referred, you will remember, was cod-liver oil in full doses, with a drink composed of cit. potass., with an excess of the acid. The character of the ulcers was changed in a very short time, and the cure was completed in a few weeks.

---

A CORRESPONDENT asks for the best remedy for Ascarides. Will some one of our contributors give us an article on that subject?

